# Recurrent Intussusception in the Setting of Meckel’s Diverticulum in an Infant

**DOI:** 10.7759/cureus.40325

**Published:** 2023-06-12

**Authors:** Stedrea Hutchinson, Abanoub Awadalla, Nicholas Pereira

**Affiliations:** 1 College of Medicine, Saint James School of Medicine, Arnos Vale, VCT; 2 Department of Pediatrics, South Texas Health System Children’s, Edinburg, USA

**Keywords:** infant intussusception, intussusception lead point, pediatric intussusception, recurrent intussusception, meckel´s diverticulum

## Abstract

Intussusception is a condition consisting of a proximal portion of the bowel contracting into a more distal bowel portion. The recurring act of intussusception is typically caused by a pathological lead point persisting within the bowel. The most common lead point for intussusception is a Meckel’s diverticulum, which arises due to the incomplete obliteration of the omphalomesenteric canal causing a true diverticulum in the small bowel. This report outlines a case of a 10-month-old male infant who experienced three intussusception episodes, eventually requiring surgical intervention. A clinician’s awareness of this phenomenon aids in implementing adequate treatment.

## Introduction

Intussusception is a common intestinal obstruction in children. It is the act of a proximal portion of the bowel telescoping into a more distal bowel portion. The persistence of intussusception is pathological due to the possibility of necrosis occurring. Intussusception happens secondary to a lead point, the most common lead point being Meckel’s diverticulum [1]. This article presents a case of a 10-month-old male infant, who came to the emergency department with multiple episodes of vomiting. A diagnosis of intussusception was made after an abdominal ultrasound was performed. After experiencing three intussusception episodes, surgical intervention was required. Meckel’s diverticulum was identified as a lead point for intussusception and removed during surgery. This unique case outlines the management of recurrent intussusception due to Meckel’s diverticulum in an infant.

## Case presentation

A 10-month-old male infant presented to the emergency department with recurrent episodes of vomiting and blood-stained “currant jelly” stools for three days. The patient presented with intolerance to food and diffuse abdominal pain upon examination. The patient’s mother had no significant labor or perinatal complications. Table 1 shows the laboratory panel completed in the emergency department.

**Table 1:** Laboratory panel completed in the emergency department

Test	Result	Reference Range
Hemoglobin	11.20 gm/dL	10.5-13.5 gm/dL
Hematocrit	33.80%	33-39%
Red blood cells	4.52 x10e6/mL	3.7-5.3 x10e6/mL
White blood cells	13.20 x10e3/mL	6.0-17.5 x10e3/mL
Platelets	379 x10e3/mcL	150-375 10e3/mcL
Sodium	135 mmol/L	132-140 mmol/L
Potassium	5.3 mmol/L	3.5-6.3 mmol/L
Chloride	101 mmol/L	97-106 mmol/L
CO2	24 mmol/L	14-23 mmol/L
Anion gap	10 mcmol/L	3-11 mcmol/L
BUN	11 mg/dL	6-17 mg/dL
Creatinine	<0.2 mg/dL	0.2-0.5 mg/dL
Calcium	9.5 mg/dL	8-11.3 mg/dL

Throughout the patient’s hospital stay, the patient underwent three abdominal ultrasounds with two ultrasound-guided gastrografin enemas. Each abdominal ultrasound expressed recurrent intussusception episodes.

**Figure 1:**
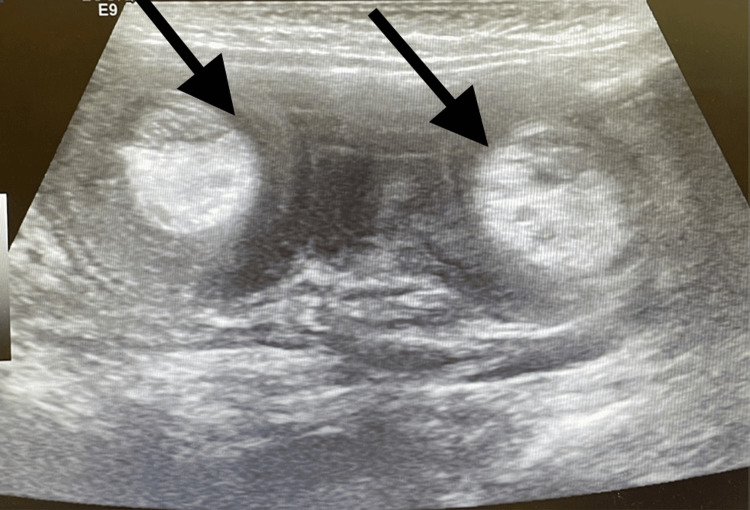
Day 1 abdominal ultrasound image showing the double target sign in the right lower quadrant

**Figure 2:**
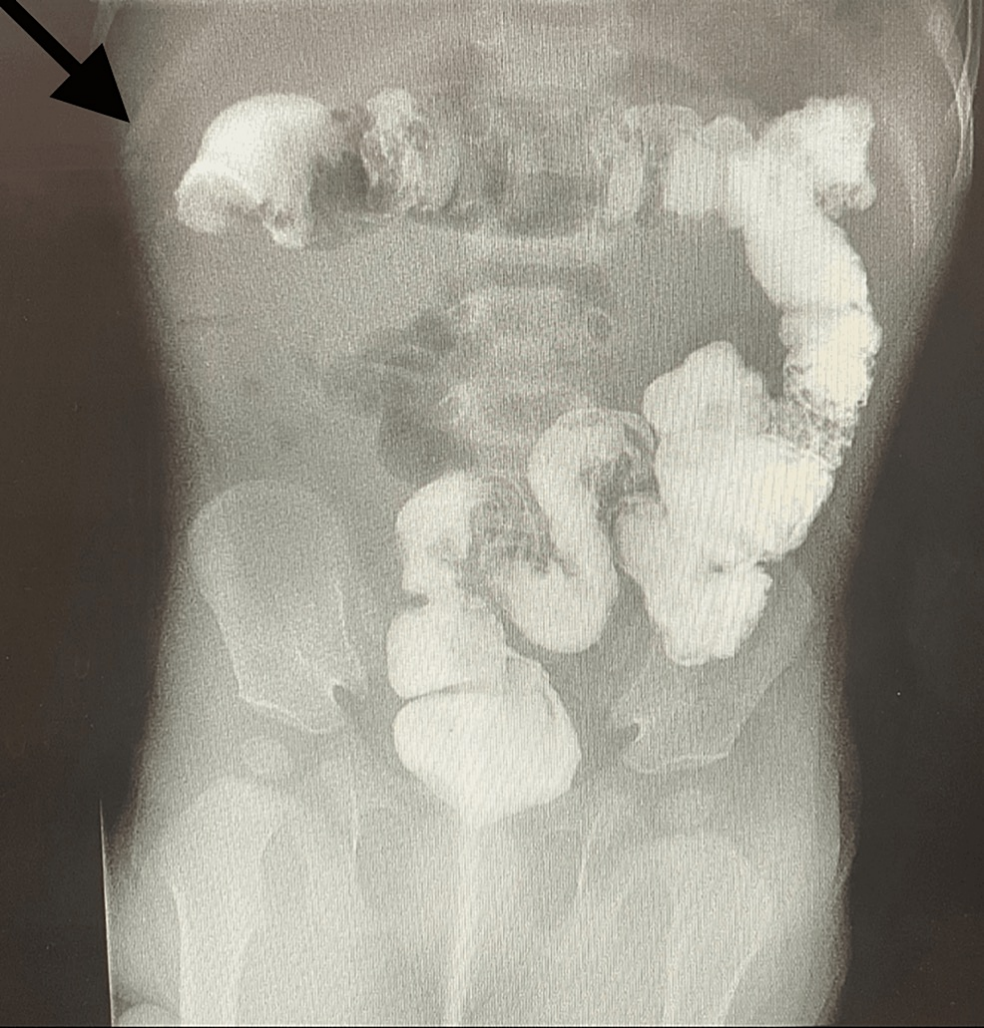
Day 1 X-ray gastrografin enema image showing the obstruction at the hepatic flexure

**Figure 3:**
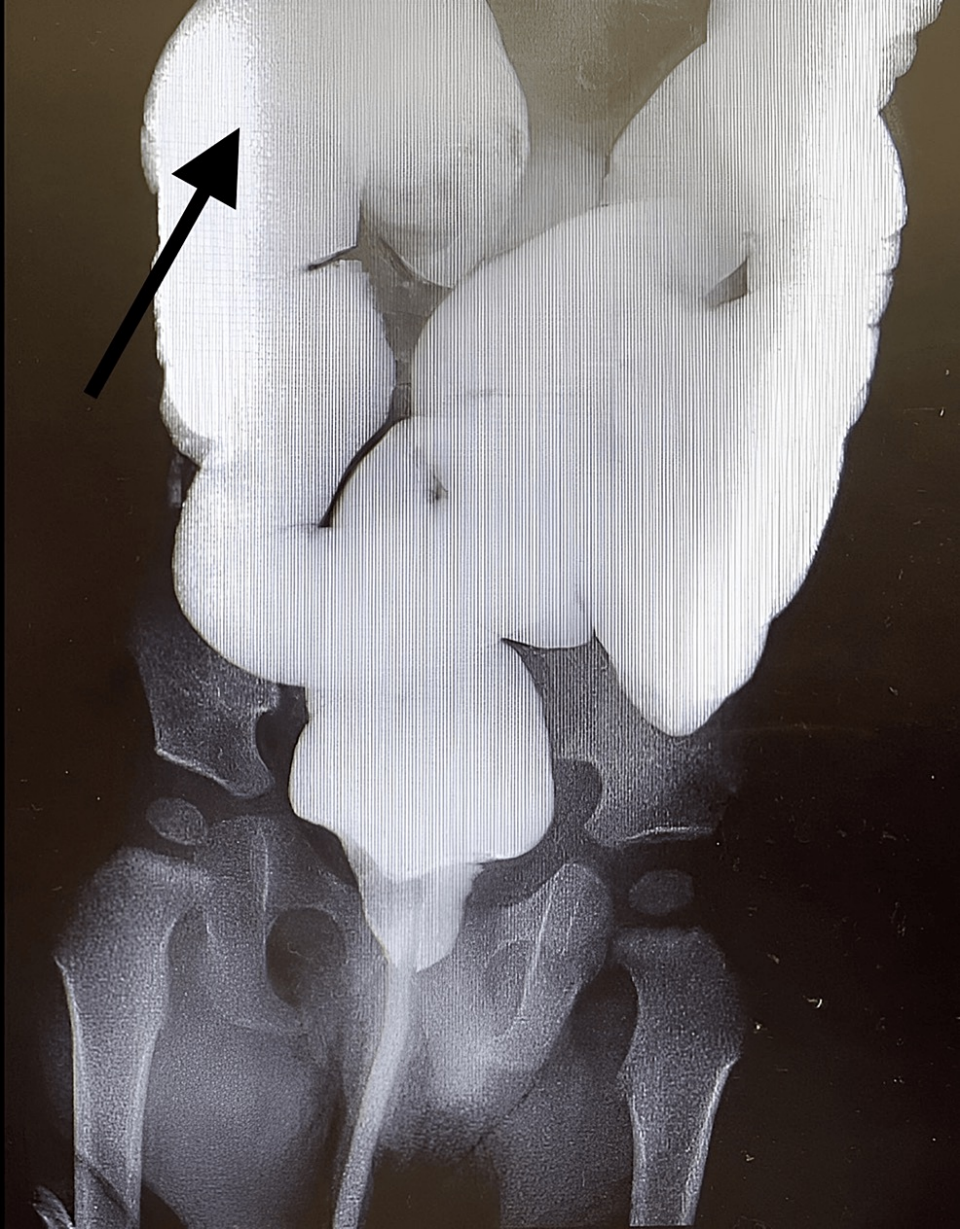
Day 1 X-ray gastrografin enema image showing the reduced obstruction

Initially, a double target sign was found in the right lower quadrant during the first abdominal ultrasound (Figure 1). A gastrografin enema was administered to the patient via ultrasound-guided imaging. The rectum, sigmoid colon, descending, and transverse colon were found to have regular opacification. Subsequently, obstruction at the hepatic flexure of the colon was noted, consistent with intussusception (Figure 2). The contrast was advanced into the ascending colon, cecum, and ileocecal valve (Figure 3). The procedure was completed without further warranted complications, and the patient was discharged on the same day with a good prognosis noted.

**Figure 4:**
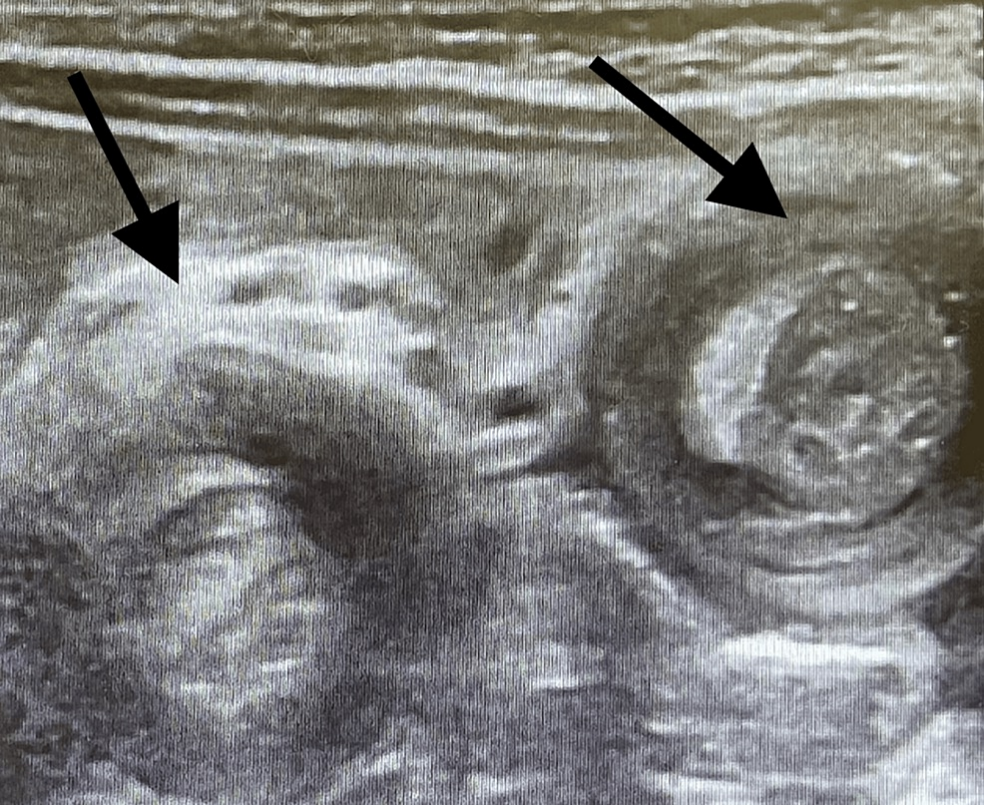
Day 2 abdominal ultrasound image showing the double target sign in the right upper quadrant

**Figure 5:**
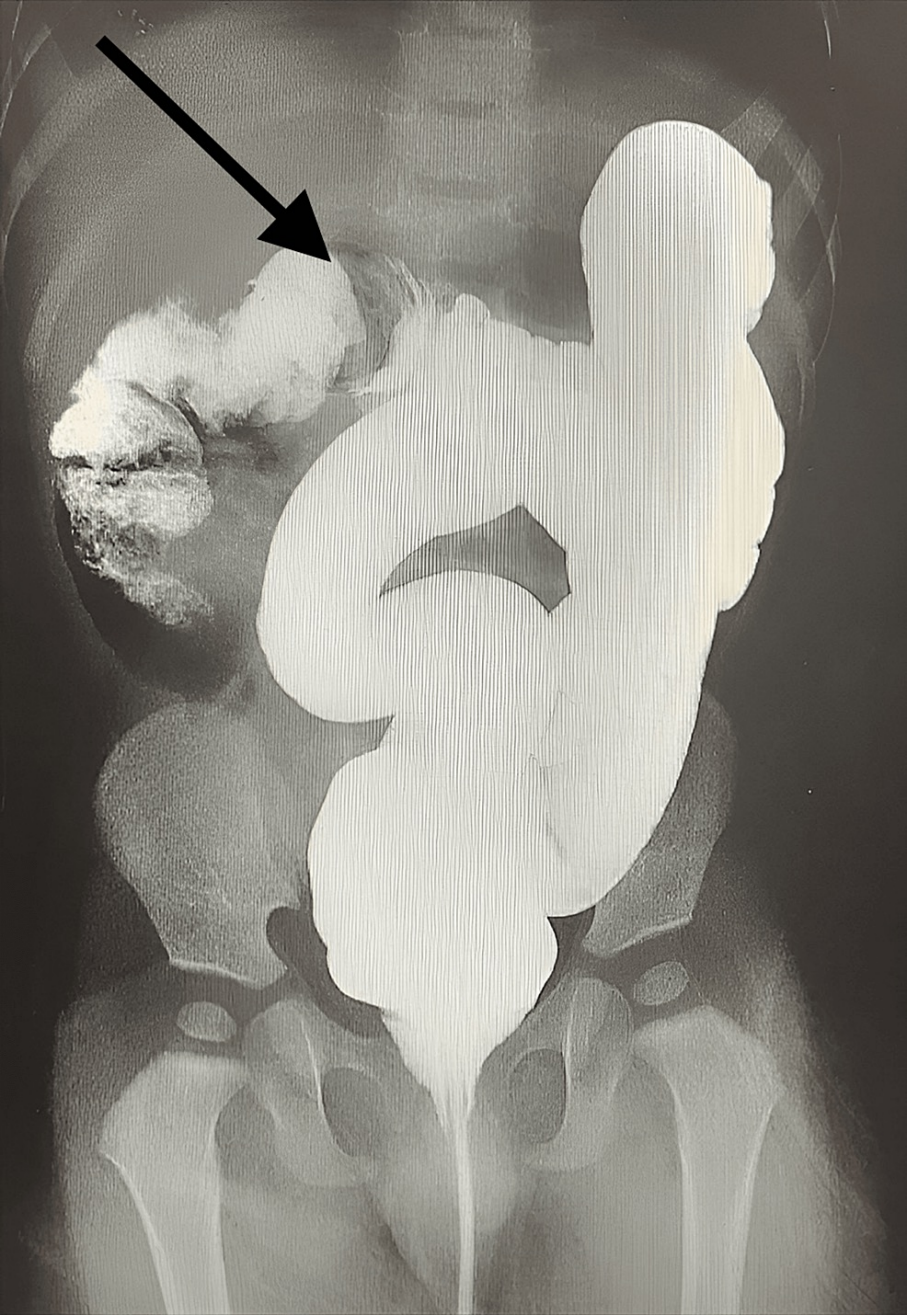
Day 2 X-ray gastrografin enema image showing the obstruction in the mid-transverse colon region

**Figure 6:**
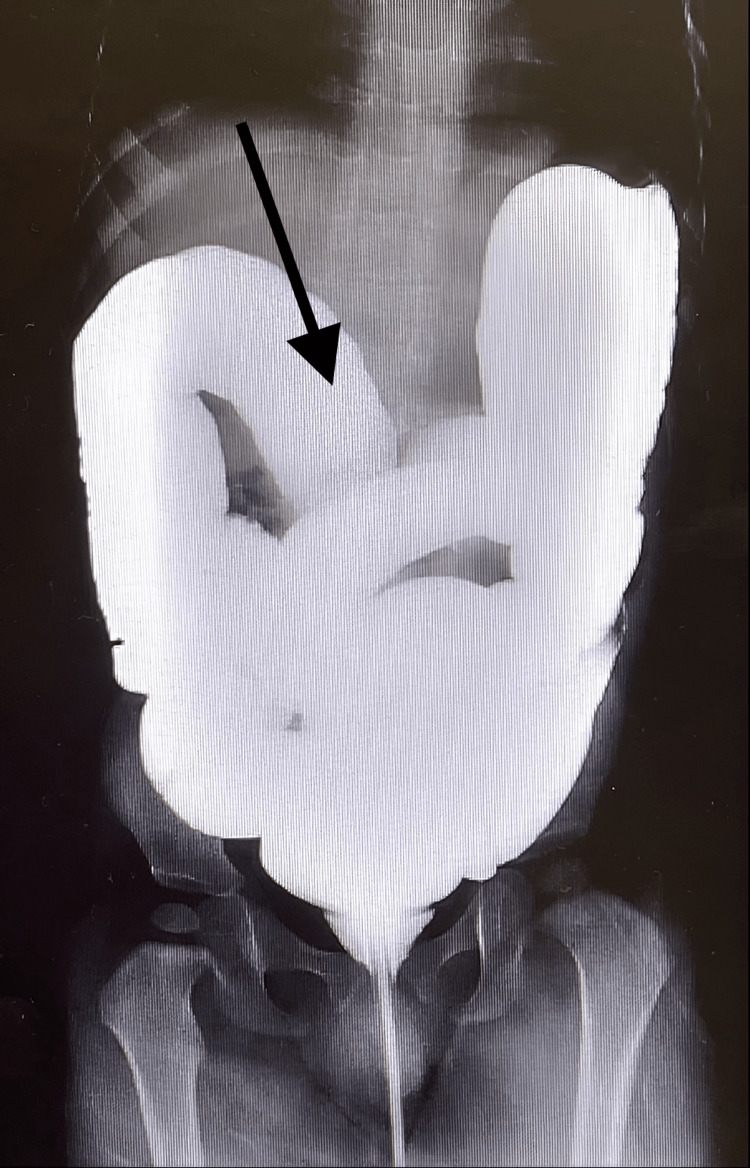
Day 2 X-ray gastrografin enema image showing the reduced obstruction

The patient returned to the emergency department the next day due to recurrent symptoms of abdominal pain, discomfort, and not feeding well at home. A repeat abdominal ultrasound was performed and indicated a double target sign in the right upper quadrant (Figure 4). A repeat ultrasound-guided gastrografin enema was performed, which indicated regular opacification of the rectum, sigmoid, and descending colon with an obstruction found in the transverse colon concluding with intussusception (Figure 5). Consequently, the intussusception obstruction was successfully reduced (Figure 6).

**Figure 7:**
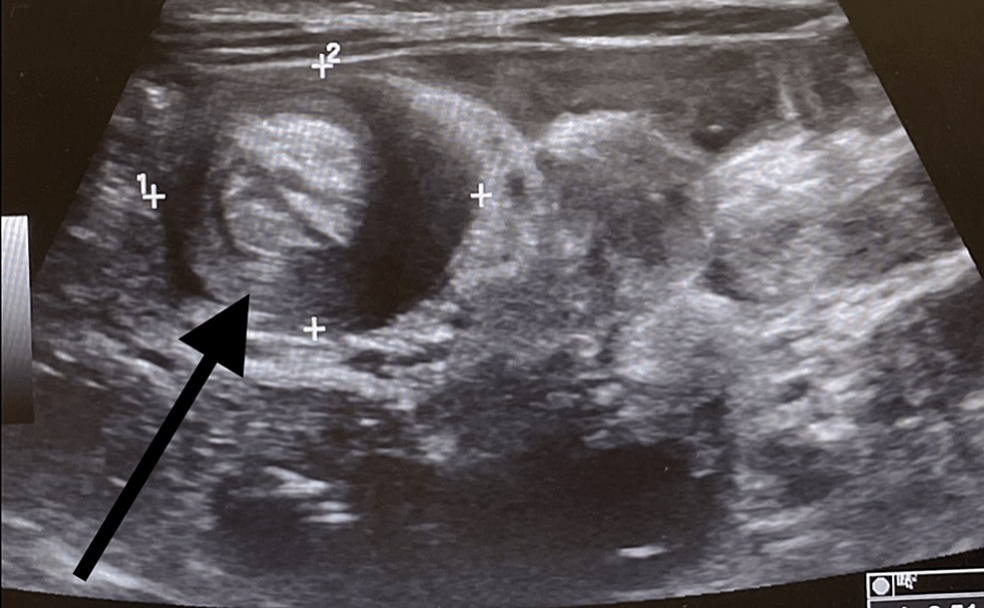
Day 3 abdominal ultrasound image showing the target sign in the right upper quadrant

Following this, the patient continued to have symptoms of recurrent intussusception such as vomiting, “currant jelly” stool, and abdominal pain. A third abdominal ultrasound was performed, which indicated a target sign in the right upper quadrant confirming a recurrent intussusception episode (Figure 7). The surgical team was consulted and it was determined that the patient was eligible for surgical intervention due to intermittent intussusception episodes.

Initially, the surgery was done laparoscopically, consisting of an initial surgical incision in the supraumbilical region, and there was no intussusception visualized in the ileocecal region. The bowel was explored proximally from the ileocecal region, in which a portion of the small bowel was intussuscepted into itself. The surgeon proceeded to manually reduce the obstruction, though no lead point was identified. The surgical team in their efforts of identifying a lead point explored the bowel starting at the ileocecal valve toward the ligament of Treitz, where a cyst was observed on the antimesenteric border of the small bowel in the ileum. The surgeon converted to an open procedure for better visualization of the cyst. Upon inspection, the cyst was concluded to be a Meckel’s diverticulum, which was invaginated internally. The surgical team selected to resect the affected bowel region including the diverticulum.

## Discussion

Intussusception can be caused by pathological leads such as Meckel’s diverticulum, lymphomas, and less commonly by polyps, duplications, and bowel wall tumors such as lipomas, hamartomas, schwannomas, lymphangiomas, and hemangiomas. In cases caused by a pathological lead, patients typically experience recurrent intussusception episodes, as exhibited by this case, due to the presence of Meckel’s diverticulum [1].

Meckel’s diverticulum is a condition resulting from the incomplete obliteration of the omphalomesenteric canal causing a true diverticulum in the small bowel. It is the most common congenital abnormality of the gastrointestinal system [2]. During embryogenesis, the yolk sac develops into two separate entities: the larger contributing to the primitive gut and the smaller evolving as a yolk sac near the placenta. These two portions remain connected via a tube called the omphalomesenteric canal or vitelline duct [3]. The persistence of the omphalomesenteric canal beyond the seventh week of development causes a Meckel’s diverticulum due to failed closure of the intestinal end of the canal [3]. Meckel’s diverticulum is considered a true diverticulum due to the presence of all three layers of the intestinal wall and occurs most frequently near the ileocecal valve on its antimesenteric border [3]. It tends to be composed of heterotopic gastric or pancreatic tissue, and rarely colonic or hepatobiliary tissue can be found, which may present with inflammation, ischemia, or bleeding [3]. The incidence of Meckel’s diverticulum is estimated to be 2% based on surgical reviews and is twice as likely in males [3].

Based on their presence, Meckel's diverticulum can cause intestinal obstruction, specifically intussusception, as presented in this case. Intussusception is an invagination of a proximal segment of the bowel into an adjacent distal portion of the bowel. It is most commonly found in the ileocecal region [1]. Peristaltic movements of the bowel against the area of intussusception promote obstruction, strangulation, and necrosis of the bowel, making intussusception an urgent matter. According to Huang et al., it was reported that 17% of Meckel's diverticulum presented as intussusception and tends to present with hematochezia [1].

Diagnosis of Meckel’s diverticulum is very challenging to common imaging modalities, such as plain X-ray, barium studies, and computed tomography scans, rarely illustrating a finding [1-2]. Ultrasonography, although not specific enough for imaging this condition, may reveal a tubular diverticulum swollen with fluid in a region away from the cecum, invagination, segmental thickening of the bowel walls, swelling of the diverticular wall, and pelvic abscess [1-2]. A diagnosis of a bleeding Meckel’s diverticulum can be made via mesenteric arteriography. All in all, technetium-99m pertechnetate scanning is the most common diagnostic tool for Meckel’s diverticulum that is non-invasive. Laparoscopy is the best diagnostic tool for definite diagnosis, especially in doubtful cases [1-2]. Intussusception caused by Meckel's diverticulum is treated by surgical resection with subsequent anastomosis, usually performed via a laparotomy or with laparoscopic assistance [1]. Intussusception due to a pathological lead point cannot be treated conservatively because reduction tends to fail; therefore, surgical laparotomy or laparoscopic assistance with resection is a definite treatment [1].

## Conclusions

In conclusion, this report summarized a case of a 10-month-old male infant who presented to the emergency department with multiple episodes of vomiting. The patient experienced blood-stained “currant jelly” stools in association with their symptoms of vomiting. During their inpatient stay, the patient expressed three intussusception incidents, diagnosed by abdominal ultrasound, two of which were reduced by gastrografin enema. The third intussusception episode was elected to be treated surgically as a definitive therapy for recurrent intussusception. During surgery, it was noted that the lead point for the patient’s recurrent intussusception was the presence of Meckel’s diverticulum. According to Huang et al., it was reported that approximately one-fifth of Meckel's diverticulum presented as intussusception, making this case an uncommon presentation. In response, Meckel’s diverticulum was exercised during surgery, which remedied the patient’s intermittent intussusception. This case report provides a great review of a unique incidence of recurrent intussusception caused by the persistence of Meckel's diverticulum. This renders healthcare providers more understanding in cases that present in a similar way.
